# The Deciduous Teeth and the Sixth Year Molars; Disastrous Results from Neglect and Their Treatment

**Published:** 1899-07

**Authors:** 


ARTICLE IV.

### THE DECIDUOUS TEETH AND THE SIXTH YEAR MOLARS; DISASTROUS RESULTS FROM NEGLECT AND THEIR TREATMENT.

Although great advancements have been made in dentistry, yet a large gap has been left which requires to be filled in. It seems strange that the deciduous teeth have received so little attention. The numerous authors who have written on diseases of the mouth, and have treated the subjects in a masterly manner, have either totally ignored the treatment of deciduous teeth, or have touched the subject very sparingly. Has this neglect been because they considered the deciduous teeth not worthy of much consideration as they last only such a short time and are replaced by a permanent set? If this is the case they have made a very grave mistake, as I consider the milk teeth act a great part in the physiology and pathology of childhood, and deserve a great deal more attention than has been bestowed upon them. Their neglect may cause serious trouble in the health of a child whose teeth have been extracted too soon or are so badly decayed as to cause pain in mastication; they cannot properly masticate their food, in consequence of which assimilation is imperfect, which causes a defective bodily development; moreover the irregularity of the permanent teeth so often met with is caused greatly by the too early extraction of the deciduous teeth. To avoid all these disadvantageous conditions there is only one remedy, viz., place the children from their early youth in the care of the dentist, and at the same time let the parents be instructed to watch their children's teeth as much as they do their own.

The eruption of the deciduous teeth is completed between the twenty-eighth and thirtieth month after birth, and from this time on, or say at three years, they should be placed in our hands. It may appear that a child of such tender years is too delicate to undergo a dental operation, from which many a grown-up person will shrink. As far as this is concerned, the child does not think about it, and if the dentist understands how to handle these delicate patients, he will have less trouble with them than he has with some of their parents. A great many parents make the sad mistake of speaking in the presence of their children about the operation and the pain they had to endure while in the dental chair. The children may not appear to be listening, but not a word escapes their attentive little ears, and when their turn comes to go to the dentist they go with a prejudiced idea.

The sensitiveness of the nerves is not so highly developed in the child as in the adult. I have found that I could touch an exposed pulp in a deciduous tooth without causing the same amount of pain as in a permanent tooth. We can therefore without much difficulty arrive at our aim, to save the milk teeth as well as the permanent from the ravages of caries, to which they so easily fall victims. We have become acquainted with the disturbances which arise in the health and normal development of children whose teeth have been left to take care of themselves. Not alone does the health suffer, but also the facial expression. The alveolar arch that has lost some of its teeth will contract rather than enlarge with the growth of the child, consequently when the permanent teeth make their appearance there is no room for them, causing irregularity, which is often very difficult to correct without regulating appliances, and sometimes the loss of one or more permanent teeth. And even if regulated the trouble does not end, as in some cases it is very difficult to retain the teeth in their new position, necessitating the wearing of retaining appliances for months or even a year or more, until ossification is complete and the space caused by moving the teeth filled up. If the retainer is taken off before ossification is complete, the teeth will have a tendency to fall back to their original position. Aside from these difficulties, if we consider how many teeth become carious through regulating appliances, we have to admit that it is of importance and necessary to keep a strict watch over the children's teeth from their earliest youth. We are well aware that a great many parents are not convinced of the necessity of this, as the general opinion is, that the milk teeth are only temporary and do not last long, therefore they require no attention. There are others who think that we dentists only fill the temporary teeth for pecuniary gain. It nevertheless remains our duty to convince the parents of the necessity of giving the children's teeth all the care and attention possible, and dentistry and humanity will profit thereby.

I will now give a short account of the remedies I employ in the treatment of carious deciduous teeth.

The treatment does not vary greatly from that of the permanent teeth, and may be divided into four groups.

1. Superficial caries where enamel and dentine is slightly effected.

2. Deeper caries, pulp not exposed.

3. Deep caries, exposed living pulp.

4. Caries, with dead pulp, abscess and fistula.

The treatment of the first and second groups is very simple; it is sufficient to excavate the cavity and fill with cement for the anterior, and cement or amalgam for the posterior teeth. In treating group three commence with the application of arsenic, for which I use Thomas' Arsenious Cotton in very small quantities and allow it to remain only twenty-four hours. This preparation does not act as deep as pure arsenic, and is less dangerous. This is then removed and the opening to the pulp chamber enlarged; if painless I apply carbolic acid to the pulp chamber and leave it for two or three days, but if painful again apply the arsenic preparation for twenty-four hours and treat with carbolic acid again. After this I extirpate the pulp, and apply iodoform and fill the cavity temporarily with gutta-percha and leave it for two or three weeks. The gutta-percha filling is used, so that in case of periostitis, it can be easily and quickly removed. Although some practitioners fill permanently immediately I prefer my method, as I have better results with it than with immediate filling. I use the precaution of a temporary filling as it is of importance to inflict as little pain as possible on the children and they will be the more ready to come for future work. The permanent fillings are the same as in groups one and two, only that the pulp chamber is filled with gutta-percha, avoiding forcing it into the canals. In reference to group four I partly clean the cavity at the first sitting, apply oil of cloves or iodoform and leave it in the cavity forty-eight hours, after which it is thoroughly cleansed. If there is an abscess it is cauterized with the galvano-cautery and an antiseptic injected into the abscess through the root. This treatment is repeated for several days, till fistula and abscess have disappeared, and a temporary filling is inserted leaving it for two or three months, and then insert a permanent filling.

This is my method of treatment. It may, perhaps, appear insignificant, but according to my idea we cannot be too circumspect in assuring ourselves of successful results of so delicate an operation.—*Zahuarztlichez Wochenblatt.*

